# Editorial: Microbial ecological and biogeochemical processes in the soil-vadose zone-groundwater habitats

**DOI:** 10.3389/fmicb.2023.1238103

**Published:** 2023-07-07

**Authors:** Huai Li, Zifang Chi, Jiuling Li, Yihao Luo

**Affiliations:** ^1^Key Laboratory of Wetland Ecology and Environment, Northeast Institute of Geography and Agroecology, Chinese Academy of Sciences, Changchun, China; ^2^Key Laboratory of Groundwater Resources and Environment, Ministry of Education, Jilin University, Changchun, China; ^3^Australian Centre for Water and Environmental Biotechnology, The University of Queensland, St. Lucia, QLD, Australia; ^4^Swette Center for Environmental Biotechnology, Biodesign Institute at Arizona State University, Tempe, AZ, United States

**Keywords:** biogeochemical processes, soil, vadose zone, groundwater, habitats

Microorganisms regulate biogeochemical cycles and serve various functions within soil, vadose zone, and groundwater habitats (e.g., Chi et al., 2018, 2022; Zhang et al., 2021; Li et al., 2022). The composition and function of these microorganisms can be influenced by both biotic and abiotic factors, which in turn affect biochemical processes and ecosystem functions (e.g., Li et al., 2019; Chi et al., 2021). Thus, there is significant interest in studying these habitats and their connection to multiple microbial pathways, particularly those involved in material cycling, pollution control, and carbon neutrality. Therefore, to develop a healthy-stable-sustainable ecosystem, this Research Topic concentrates on microbial ecological/biogeochemical processes in the soil-vadose zone-groundwater habitat. The objectives of this Research Topic are: (1) to compile new research on microbial ecological processes in these habitats; and (2) to highlight the possibilities for achieving sustainable processes. The articles included in this Research Topic have undergone careful review, and the following eleven articles have been accepted.

Zheng et al. conducted a comparative analysis of bacterial communities under different chemical oxygen demand to nitrogen (COD/N) and nitrogen to phosphorus (N/P) ratios in restored wetlands. They discovered that variations of nitrogen source controlled the bacterial composition, while imbalance in organics and nutrients resulted in bacterial community differentiation.

Yan et al. investigated the impact of various remediation measures on the removal of ${\text{SO}}_{4}^{2-}$, Pb, Zn, and Mn in rare earth mine (REM) soils. The results indicated that chemicals (Ca(OH)_2_, 3.0 g/kg) plus sulfate-reducing bacteria (SRB) (CM-M) exhibited greater efficiency in removing of ${\text{SO}}_{4}^{2-}$, Pb, Zn, and Mn. The inoculation of SRB was beneficial for increasing sulfur and nitrogen cycling. This study provided a good method for REM contaminant removal.

Kuang et al. identified Proteobacteria and Actinobacteria as the dominant phyla in Baiyangdian sediment under eutrophication. These phyla actively participated in C, N, P, and S cycles. The genes associated with these cycles were correlated with the presence of the reductive-citric acid cycle pathway. Carbon-metabolism was dominant for the bacterial community. The abundances of genes related to nitrogen cycle were consisted with high total nitrogen (TN) level.

Wang M. et al. investigated the response of soil property, enzyme activity, and bacterial community to different hydrological practices in the Changbai Mountains. Their findings revealed that a high water level promoted the recovery of soil nutrients and bacterial activities and communities. These results provide valuable guidance for implementing effective strategies to restore peatlands.

Zhao et al. explored the effect of soil depth and altitude gradients on microbial abundance in the Changbai Mountain. They determined that soil depth, rather than altitude, served as the primary controlling factor. Consequently, changes in soil depth may lead to greater disturbances for microorganisms in the face of future climate change.

Song et al. examined the influence of rising temperature and adding nitrogen on CO_2_ and N_2_O emissions and microbial abundances in permafrost-peatlands. They observed that increased temperature, nitrogen availability and their combined effect remarkably increased CO_2_ and N_2_O emissions. These findings demonstrated that soil microorganisms and available nitrogen were favorable for controlling carbon emissions.

Chen et al. presented a study on the benefits and drawbacks of two methods for removal of antibiotic resistance genes (ARGs) from soil. They highlighted how constructed wetlands could regulate ARG removal by utilizing different plants, substrates, wetlands, and hydraulic conditions. Photocatalysis, facilitated by catalysts and radiation intensities, effectively deactivated ARGs by producing reactive oxygen species. Combining constructed wetland with photocatalysis technology was feasible for ARG removal.

Wang Y. et al. explored antibiotic removal and biological response of different constructed wetlands. Their findings demonstrated that a combination of gravel substrate and algal/bacteria communities could effectively enhance the antibiotic wastewater removal, and maintain harmonious biological communities.

Zhang, Bai, Zhang et al. investigated the response of bacterial communities in rhizosphere and non-rhizosphere sediments of reed in Baiyangdian Lake under antibiotics stress. They found that total antibiotics and ciprofloxacin played a dominant role in regulating bacterial diversity. Antibiotics dramatically affected bacterial community composition and had a potential risk for the dissemination of ARGs in shallow lakes.

The same authors (Zhang, Bai, Zhai et al.) further explored the characteristics of bacterial communities in wild and cultivated reed zones. Their results indicated that antibiotic pollution resulting from planting activities had significant impacts on the bacterial community. These findings provide useful references for managing antibiotics in lake systems.

Liang et al. conducted a comprehensive summary of microbial communities in coastal wetlands, highlighting their controlling factors. They also discussed patterns of functional genes, revealed environmental functions, and proposed future directions for research in this field. These findings offer valuable insights into the potential application of microbes in material circulation and pollution remediation.

We think that all accepted articles in this Research Topic will provide new knowledge on microbial processes in soil-vadose zone-groundwater habitats.

## Author contributions

All authors listed have made a substantial, direct, and intellectual contribution to the work and approved it for publication.
